# Establishment and equilibrium levels of deleterious mutations in large populations

**DOI:** 10.1038/s41598-019-46803-7

**Published:** 2019-07-17

**Authors:** Johan W. Viljoen, J. Pieter de Villiers, Augustinus J. van Zyl, Massimo Mezzavilla, Michael S. Pepper

**Affiliations:** 1 Development, Research and Technology Department, Hensoldt Optronics, Centurion, 0157 South Africa; 2 Department of Electrical, Electronic and Computer Engineering, EBIT, University of Pretoria, Pretoria, 0028 South Africa; 3 Radar and Electronic Warfare Research and Applications Group, Council for Scientific and Industrial Research, Pretoria, 0001 South Africa; 4 Department of Mathematics and Applied Mathematics, University of Pretoria, Pretoria, 0028 South Africa; 5 Institute for Maternal and Child Health, IRCCS ‘Burlo Garofolo’, Trieste, Italy; 6 The Wellcome Trust Sanger Institute, Wellcome Genome Campus, Hinxton, Cambridgeshire CB10 1SA UK; 7 Institute for Cellular and Molecular Medicine, Department of Immunology, and SAMRC Extramural Unit for Stem Cell Research and Therapy, Faculty of Health Sciences, University of Pretoria, Pretoria, 0084 South Africa

**Keywords:** Computational models, Experimental models of disease

## Abstract

Analytical and statistical stochastic approaches are used to model the dispersion of monogenic variants through large populations. These approaches are used to quantify the magnitude of the selective advantage of a monogenic heterozygous variant in the presence of a homozygous disadvantage. Dunbar’s results regarding the cognitive upper limit of the number of stable social relationships that humans can maintain are used to determine a realistic effective community size from which an individual can select mates. By envisaging human community structure as a network where social proximity rather than physical geography predominates, a significant simplification is achieved, implicitly accounting for the effects of migration and consanguinity, and with population structure and genetic drift becoming emergent features of the model. Effective community size has a dramatic effect on the probability of establishing beneficial alleles. It also affects the eventual equilibrium values that are reached in the case of variants conferring a heterozygous selective advantage, but a homozygous disadvantage, as in the case of cystic fibrosis and sickle cell disease. The magnitude of this selective advantage can then be estimated based on observed occurrence levels of a specific allele in a population, without requiring prior information regarding its phenotypic manifestation.

## Introduction

A computational and statistical framework was created to simulate and calculate the diffusion of monogenic variations over multiple generations through a population of diploid organisms. Although the work was originally aimed at exploring aspects of cystic fibrosis (CF) in humans, the tool is generally applicable to the modelling of the epidemiology of monogenic variants, where there may be differences in fitness and/or survival success rates between homozygous and heterozygous individuals. This is based on the observation that heterozygous carriers of some common mutations, e.g. in the cystic fibrosis transmembrane conductance regulator (CFTR) gene, seem to have a survival/fecundity advantage compared to wild-type individuals^1,2^, while the homozygous state results in a definite disadvantage. An example of a heterozygous advantage that is environmentally dependent is malaria resistance as a consequence of specific mutations in the haemoglobin gene^3,4^, bringing with it the risk of sickle-cell anaemia in homozygotes. Another common example of a heterozygous advantage is the major histocompatibility complex (MHC) in vertebrates^5^. We also investigate the establishment behaviour of purely beneficial variants, such as the one linked to lactase persistence, which can confer an advantage on homozygotes as well as heterozygotes, depending on the nutritional environment.

The implications of such a benefit (termed ‘selective advantage’) were investigated by Haldane^6^, who focused on the mathematical probability of purely beneficial variants becoming established in a population of size *N*. Haldane’s results were subsequently refined by Wright, Kimura, and others^7–10^. Wright also introduced the notion of ‘effective population size’ (*N*_e_), to account for effects such as non-random mating, inbreeding and unequal sex ratios, which may influence the effectiveness of natural selection forces.

For human populations we further extend this approach by drawing on the results of Dunbar and Lehmann to propose a realistic range and upper limit to the size of the group from which an individual is likely to select a mate^11,12^. This is analogous to the ‘breeding unit’ or ‘neighbourhood’ introduced by Wright^13^ in 1946, being the spatially closest individuals in a circular area with a radius of 2*σ*:

1 $${N}_{{\rm{n}}}=4\pi {\sigma }^{2}d$$

with *σ* being the standard deviation of a spatially distributed 2-dimensional normal distribution around an individual and *d* the distribution density. This reflects the observation that the parents of an individual organism are more likely to be proximate than remote, and, as Nunney^14^ suggests, that *N*_n_ will be relatively constant (unlike *N*_e_), subject to the assumptions that *σ* is a property of the species and that there is a negative correlation between dispersal and density, i.e. that one can normalize for *d*. This leads to a parental probability distribution solely dependent on distance *r*:

2 $$f(r)=\frac{1}{\sqrt{2\pi {\sigma }^{2}}}{e}^{-\frac{{r}^{2}}{2{\sigma }^{2}}}.$$

In the case of human populations, we propose that the concept of distance (*r*) should be reinterpreted as social proximity, rather than necessarily physical proximity, due to the global mobility (i.e. potentially high dispersal) that has been attained by humans in recent times, and which does not necessarily translate into an increase in *N*_n_ (which is also the pool from which an individual would usually select a mate).

The effective population size *N*_e_ is the size of a virtual population that would have the same stochastic properties as the actual population under consideration. This is usually estimated from genetic variation, using variations in linkage disequilibrium patterns (using single nucleotide polymorphisms), or using information from whole genome sequences. For human populations *N*_e_ tends to be variable, but in general it is significantly larger than *N*_n_. Li and Durbin^15^ estimate the current *N*_e_ for various human subpopulations to range from about 9,000 to 50,000, with some historical bottlenecks as low as approximately 1,000 individuals.

In their work performed on the genetics of guinea pigs and fruit flies, Wright and Dobzhansky^13,16–18^ additionally had to introduce an inbreeding factor *F* and an immigration index *m*. This is required to compensate for correlation between uniting gametes, and the postulated expectation that ‘immigration’ is likely to be local, from adjacent localities. These areas will probably resemble the target locality (in a gene frequency sense) rather than that of the entire species, which immediately imposes yet another estimated adjustment^18^ to obtain an *effective* value for *m*. We propose a simpler neighbourhood definition for humans (in which self-fertilization can also be neglected), where breeding structure is abstracted to the social dimension, and inbreeding, migration, and genetic drift become emergent features, rather than modifying requirements to approximate actual observations.

Based on primate studies and the size of the human neocortex, Dunbar^11^ posits a nominal maximum group size of 148 for humans (usually rounded up to 150), with a 95% confidence interval between 100 and 230, but also notes that this is an upper value, which is only approached under extreme environmental stress, where the significant time and energy investments of maintaining close social bonds are repaid by the survival benefit realized by being part of a larger group. In more prosperous times, group sizes tend to reduce. Wright and Nunney’s Gaussian neighbourhood definition as in Eqs (1) and (2) is therefore applied to humans, in social space, with the size of each individual’s network of close social relationships conservatively constrained by Dunbar’s number. MacCluer and Schull^19^, using a crude stochastic model to recreate observed gene frequencies in humans, similarly estimate the effective population size for human populations to range between 65 and 200, depending on several assumptions.

The domain of the normal distribution is infinite, and therefore includes the entire finite population *N*. This means that while breeding between proximate individuals is far more likely, there is always a non-zero probability that two remote individuals may interact. Breeding normally requires physical proximity, and hence such an eventuality implies that migration can and does occur. The immigration index *m* and inbreeding factor *F* are therefore not independent, but related via the standard deviation *σ*. The exact relationship is of course likely to be complex, and beyond the scope of this investigation. Nonetheless, it will be shown that known observed gene frequencies in human populations can be modelled by merely adjusting *σ* within the range as proposed above.

For modelling purposes, a two-dimensional grid lends itself well to processing and visualisation; this does not imply that human social networks (of the close, meaningful kind, as described by Dunbar, and specifically not the far more tenuous constructs found on social media) are necessarily two-dimensional in nature. However, the only relevant characteristic of the distribution is the distance *r* as in Eq. (2). Irrespective of the dimensionality of the grid, the correlation between the Gaussian parental probability distribution functions of two individuals depends solely on the distance *r* between them, scaled by the standard deviation *σ*. This correlation function, being the convolution of two normal distributions, is of course itself a normal distribution, with twice the variance of the parental probability function.

This aspect is one of the major contributions of our framework, which allows us to simulate large populations (*N*) while modifying the community size (*N*_n_) in order to create scenarios to study how local selection affects the pattern of distribution of deleterious variants.

We then explore the effect of this community size, in conjunction with the selection coefficient of a given variant, on the probability that such an allele will become established in the population, and on the eventual equilibrium levels that are reached, especially in the case of variants that are beneficial when heterozygous, but pathogenic (or less beneficial) when homozygous. Such alleles do not necessarily become ubiquitous once established, a possibility that Wright and Dobzhansky erroneously dismissed in their seminal work on lethal mutations in fruit flies^20^. Conversely, if we know the occurrence levels of such a variant in a population, we can use our model to estimate the selective advantage that it confers on heterozygotes, without requiring any knowledge of the specific manifestation and mechanism of such a selective advantage.

## Establishment of Variants

For diploids, the selection coefficient *s* associated with a specific variant is defined as an additive term, such that heterozygotes would, on average, have (1+*s*) times as many offspring as wild-type individuals^21^. Note that *s* can also be a negative number, implying a disadvantage by resulting in fewer offspring of mutant heterozygotes, whether through lowered fertility, or decreased survival to procreative age (which in some sense is the same thing - irrespective of the mechanism, the result being a reduction in the number of offspring compared to wild-type individuals). Although the case of *s* < 0 has the physical interpretation of a selective *disadvantage* (purifying selection), the case of *s* < −1 is meaningless, and as such the parameter *s* should be constrained on the interval [−1, ∞).

Using a deterministic model, any beneficial variant (that is, with *s* > 0) will inevitably grow in prevalence, guaranteeing eventual fixation of the advantageous allele in the population. However, in reality genetic drift causes random fluctuations in the frequency of lineages, which can easily extinguish even highly beneficial alleles when its prevalence is low, as will be shown. A stochastic treatment is used to analyse such situations, which especially apply whenever a new mutation appears *de novo* in a single individual. The allele will only be established in the population (and only then a deterministic model may be applicable) if this variant survives genetic drift. During admixture between different populations, ‘new’ alleles are introduced at significant levels into both groups. Under such conditions (relatively large populations and high prevalence) the alleles could be considered to already be established and therefore to be less subject to the vagaries of genetic drift, but rather with their eventual fate dominated by the relative selection coefficients that the alleles confer (i.e. closer to the deterministic case).

Addressing the fixation probability *P* of a single copy of an advantageous allele in a large population, Haldane found that $P\approx 2s$ if *s* is small^6^. Barrett *et al*.^22^, drawing on this as well as on the subsequent analyses for finite populations by Kimura^7,9^, show that

3 $$P\approx 1-{e}^{-2s}$$

which also accurately approximates the probability that a single advantageous allele with a large positive value of *s* will survive stochastic loss. Negative or zero values of *s* always lead to eventual extinction. Migration within the population under consideration has no effect, as panmixia is implicitly assumed, which implies a homogeneous landscape, whether it be geographic or social, and therefore transplanting an allele does not impact its viability.

## Results

### Stochastic determination of fixation probability

**Figure 1:**
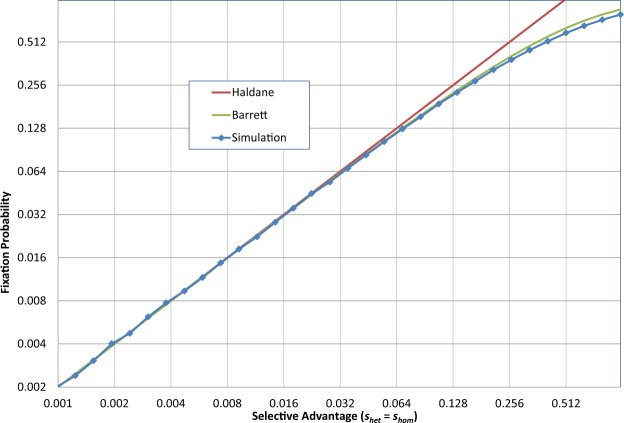
Establishment probability of a beneficial allele. Fixation rate for a single variant as a function of (positive) selection coefficient (*N* = *N*_n_ = 2.5 × 10^5^). (See File S1).

To investigate the statistical fate of a dominant variant with a positive selection coefficient *s* (for both heterozygous and homozygous cases) in a large population, our simulation tool was configured to introduce a single variant in a virtual population (population size *N* = 2.5 × 10^5^), and then to cycle through the generations until the allele either becomes ubiquitous or extinct. This was repeated 200,000 times for each point in the graph shown in Fig. 1, for a total of ~112 million generations. The results confirm Haldane’s prediction, including his *caveat* that it is only valid for small values of *s*. The stochastic simulation initially closely follows the *P* = 2*s* line, but then gradually deviates from it as *s* increases, as predicted by Barrett’s approximation in Eq. (3). This is expected: the fixation probability cannot exceed 1; it can only approach unity asymptotically as *s* grows.

### The price of success

- *Homozygosity:* A variant that is purely beneficial will completely displace the wild-type allele only if it successfully runs the gauntlet of genetic drift while still rare. In a diploid population, however, heterozygotes may derive a selective advantage from a given variant, while homozygosity results in a reduced selective advantage (or even disadvantage), such as in the case of CF, sickle-cell disease and others. As the prevalence of such an allele in a population increases, the probability of producing homozygous offspring also rises, to the point where the relative disadvantage of homozygosity exactly balances the heterozygous advantage. An equilibrium is reached, depending on the relative magnitudes of the effects, as well as the population parameters, especially the effective population size *N*_e_ and the neighbourhood size *N*_n_.
- *Environmental factors:* The striking geographic correlation between the distribution of the sickle-cell allele and the prevalence of malaria^4^ demonstrates this effect. As long as the local population is exposed to malaria, the allele (if present) confers an advantage (*s* > 0) to heterozygotes and rises to prevalence levels limited by the negative effects caused by the associated increase in homozygous individuals. Where malaria is absent, there is no selective advantage (*s* may even be slightly negative), and the allele becomes extinct. Environmental variables definitely matter.
- *Migration, selection and inbreeding:* A shortcoming of Haldane’s approach is that the population *N* is assumed to be large, constant and with equal sex ratios and random mating. This is not normally the case. Many subsequent researchers have addressed this^10,21,23^ and have introduced the concept of an effective population size *N*_e_ which would result in the same variance as the actual population in question. Usually the effective population size is smaller than the census size (*N*_e_ < *N*), and there is the even smaller community size (or neighbourhood number) *N*_n_ which affects genetic differentiation between subpopulations: the smaller *N*_n_ the larger the differentiation between them, due to the decreased dispersal distance and increased genetic drift, considering limited or no gene flow^14^.

### Equilibrium in the absence of a Dunbar limit

To appreciate the effect of the Dunbar limit, it is instructive to recall the case of purely random mating in the absence of such a limit. Equilibrium under selection for alleles of two types was studied by Fisher^24^. We rephrase his result in our terminology. Consider a population in which the ratio *homozygous:heterozygous:wild* is given by P:2Q:R. Fisher shows that if by selection the ratio for the parents is ${a}{\rm{P}}:2{b}{\rm{Q}}:{c}{\rm{R}}$, where *a*, *b* and *c* are constants so that ${a}{\rm{P}}+{b}{\rm{Q}}+{c}{\rm{R}}=1$, then under purely random mating equilibrium is only possible if ${\rm{P}}={{p}}^{2}$, ${\rm{Q}}={pq}$ and ${\rm{R}}={{q}}^{2}$, where ${p}={a}{\rm{P}}+{bQ}$ and ${q}={b}{\rm{Q}}+{c}{\rm{R}}$. Since we are interested in ratios only, we can assume $\,{\rm{P}}+2{\rm{Q}}+{\rm{R}}=1$. Then ${p}+{q}=1$ and *p* is the fraction of variant alleles.

In the case of CF in humans, the effective homozygous selection coefficient *s*_*hom*_ → −1 (at least until very recently), hence ${a}=0$. Heterozygotic advantage of *s* over the wild type implies that ${b}=(1+{s}){c}$. From this,${p}={a}{\rm{P}}+{bQ}$, and ${q}={b}{\rm{Q}}+{c}{\rm{R}}$ as above, one can show that ${p}={s}/(1+2{s})$. The fraction of heterozygous carriers is

$$2{\rm{Q}}=2pq=2\frac{s}{1+2s}\frac{1+s}{1+2s}=2\frac{s(1+s)}{{(1+2s)}^{2}}.$$

For small *s* this fraction can be approximated by

4 $$2Q=\frac{2s}{(1+4s)}.$$

**Figure 2:**
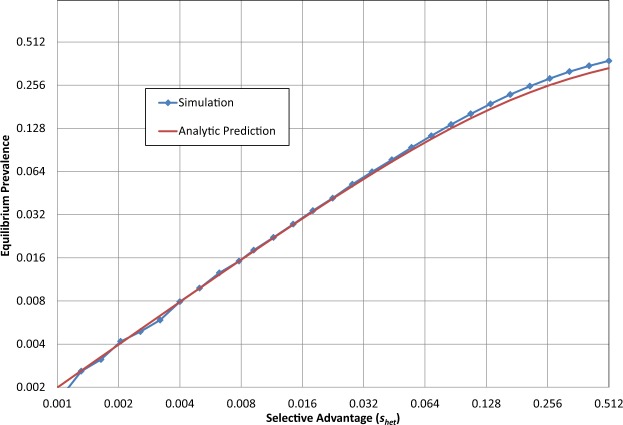
Equilibrium levels of a deleterious allele. Equilibrium level as a function of (positive) heterozygous selection coefficient, with homozygous selection coefficient = −1 (*N* = *N*_n_ = 9 × 10^6^, *s*_*hom*_ = −1, generations = 5,000). (See File S1).

As before we now compare our analytic equilibrium prediction in Eq. (4) with our stochastic simulation (setting *N*_n_ to a large value, *s*_*hom*_ = −1, and varying the heterozygous selection coefficient *s*_*het*_), achieving correspondence as shown in Fig. 2, where for small *s* (and resultant small *p*) the results are essentially identical, as expected.

### Simulation Results

Having confirmed accurate modelling of the edge cases of the well-known establishment probability (Eq. (3)) and equilibrium levels (Eq. (4)) under panmictic conditions, the computational tool described above was then used to explore the behaviour of different types of monogenic variation under various conditions. Firstly, the equilibrium behaviour of two well-studied alleles in humans are modelled. Cystic fibrosis, which is highly deleterious in its homozygous state, nonetheless confers a positive selective advantage on heterozygous individuals, while lactase persistence seems to be advantageous in both states. These equilibrium analyses are only concerned with the equilibrium behaviour, and hence establishment is implied in these cases. The model was configured accordingly for these two conditions, and run for hundreds of generations, until equilibrium levels similar to those known to be present in humans was reached. Then a series of Monte Carlo runs were executed to investigate the expected establishment and equilibrium behaviour of recessive deleterious variations while varying both the heterozygous selection coefficient and the community size over several orders of magnitude.

### Single Runs

**Figure 3:**
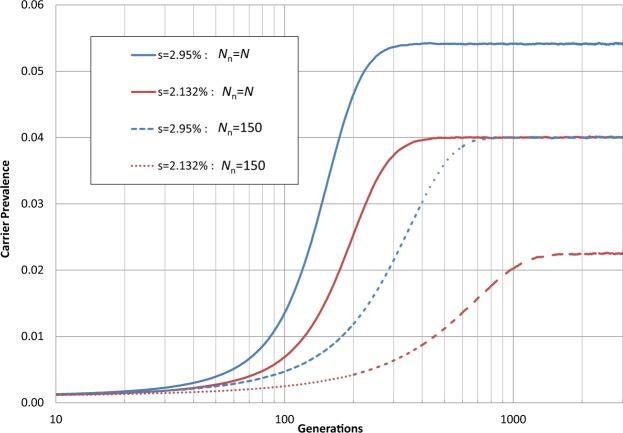
CFTR Carrier prevalence. Results for numerical simulations of allele dissemination in a test case to match a 4% CFTR allele carrier frequency. *N* = 10^8^, with each curve the average of four independent simulation runs to 3,000 generations. (See File S1).

*Homozygous deleterious (cystic fibrosis)*: Figure 3 was generated to test the numerical simulation, using two different selective advantage values *s* for heterozygotes carriers, with homozygotes having a 100% disadvantage (i.e. none survive to procreate, which approximates reality for CF). To stabilize at a prevalence of 4% (reflecting the assumed approximate CF carrier frequency in the European population) requires either a large community size *N*_n_ and *s* = 2.132%, or, more realistically, a community size of 150 (approximately Dunbar’s number for humans) and *s* = 2.95%. Both of these scenarios eventually reach equilibrium at 4%, after hundreds of generations.

The implication is that the actual advantage of being a heterozygote is indeed higher than the result that would be obtained without taking into account the limited community size, where consanguinity (or small community size) augments the probability of producing homozygous offspring.

*Positive selection*: Lactase persistence is an autosomal-dominant inherited genetic trait^25–27^ associated with the LCT gene (MIM 603202) and is especially prevalent in Northern European populations, with evidence of strong recent selection during the last 5,000–10,000 years^28^, coinciding with the domestication of cattle and a rise in dairy farming. In such a setting, the ability of adults to derive nutrition from a dairy-based diet confers an obvious advantage.

**Figure 4:**
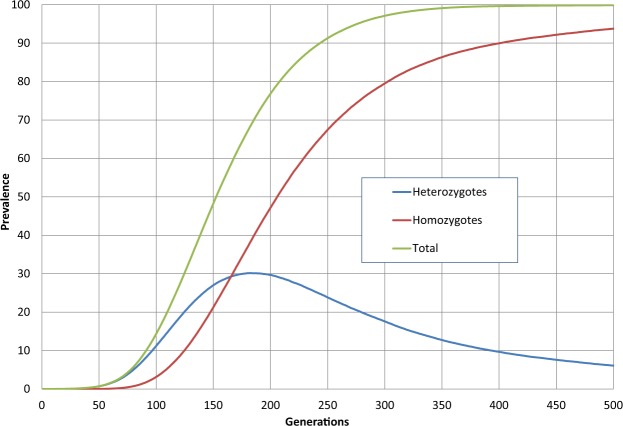
Lactase persistence. The spread of a purely beneficial allele (*s*_het_ = *s*_hom_ = 0.1, *N* = 5 × 10^6^, *N*_n_ = 150). (See File S1).

Bersaglieri *et al*.^28^ estimate the selection coefficient for lactase persistence to be between 0.09 and 0.19 for the Scandinavian population (where the prevalence of the −13910T allele currently exceeds 80%). In Fig. 4 a conservative value of 0.1 (10%) was assumed as the selection coefficient for both heterozygotes and homozygotes. With such a purely advantageous variation, and assuming, as here, that establishment has already taken place, the allele will inevitably grow to eventually completely displace the wild type, given enough time. We observe the temporal behaviour of this displacement process as the model steps through the generations. Starting from an initial low base, the prevalence of heterozygotes grows to a maximum value of just over 30%, by which time it has already been surpassed by homozygotes, which asymptotically approach 100%, given enough time (generations). After just more than 200 generations, 80% of the population carries the allele – at a nominal 29 years per generation^29^ this corresponds to 5800 years, which is near the lower end of the antiquity estimate for dairy farming. However, it seems reasonable to assume that dairy farming also took a long time to become common, which implies that the availability of milk, and hence the effective selection coefficient on LCT, gradually increased to its current value.

### **Monte Carlo Analyses**

A Monte Carlo analysis was performed in which multiple runs were executed automatically while varying the starting conditions and input parameters. This enabled the compilation of statistical results over millions of trials.

**Figure 5:**
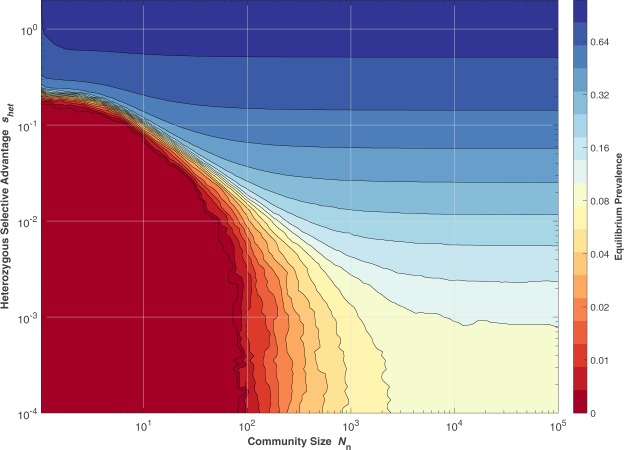
Equilibrium prevalence percentage as a function of Community Size and Heterozygous Selective Advantage. Monte Carlo run results (*N* = 10^6^, *s*_*hom*_ = −1, max 10,000 generations per point). Colour-deficiency friendly hue mapping^33^. (See File S2).

*Equilibrium levels*: Figure 5 illustrates how the eventual equilibrium prevalence of a variant (with homozygous selection coefficient *s*_*hom*_ = −1) depends on both the community size and the heterozygous advantage that it confers. Figure 2, presented earlier, is valid for large values of *N*_n_ and therefore corresponds to a vertical cross-section of Fig. 5 on the right-hand side. To reduce stochastic noise a large *N* is used, and multiple runs are averaged. Fixation is assumed: these simulations do not include recurrent mutations and the associated drift survival probabilities. Because *N*_n_«*N*, there is significant differentiation between subpopulations due to decreased dispersal distance and increased genetic drift, as noted by Nunney^14^. This leads to random local fluctuations in the gene frequencies, which statistically reduce the average prevalence level. When, due to stochastic effects, the prevalence of the variant locally exceeds the expected equilibrium value, the average incidence of homozygous individuals also increases in the same locality, resulting in an immediate downward influence on the local allele prevalence, due to the highly deleterious *s*_*hom*_ = −1. Conversely, when the local prevalence stochastically varies to below-average values, or even becomes locally extinct, it has to be ‘recolonised’ from adjacent areas, which can take many generations, especially when *N*_n_ is small. In other words, local prevalence peaks are rapidly evened out, while valleys can take a long time to fill.

**Figure 6:**
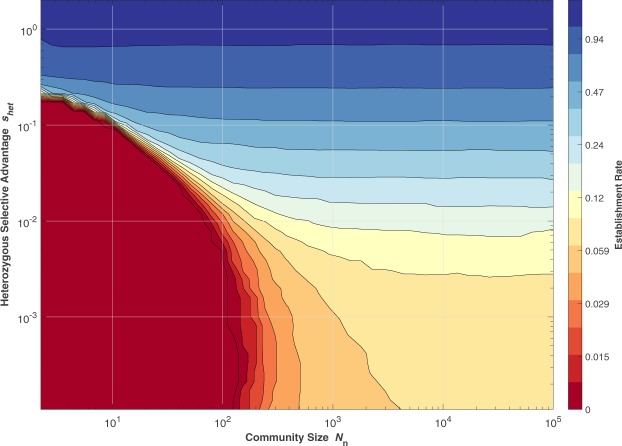
Rate of establishment as a function of community size and heterozygous selective advantage. Monte Carlo run results (*N* = 10^6^, *s*_*hom*_ = −1) (See File S3).

*Establishment*: Figure 6 shows the probability that a single allele will indeed become established, once again as a function of community size and the heterozygous selective advantage that it confers, while keeping the homozygous selection coefficient equal to −1, which approximates the case found in diseases such as CF. When the community size is large, the behaviour approaches that seen in Fig. 1, which, as above, represents a vertical cross-section through Fig. 6 on the right-hand side. From the data generated by the simulations underlying this plot one can also extract statistics regarding the average survival (in generations until extinction, if this happened) and maximum prevalence that a given variant attained.

## Discussion

In this study, we have addressed the establishment behaviour and equilibrium characteristics of monogenic variations in diploid populations, using a stochastic model with breeding interactions abstracted to a normal distribution in social space.

We found that the ability of the stochastic simulation framework to recreate known theoretical analytic results, regarding establishment probabilities of various types of variations, as well as equilibrium levels in the cases where establishment has occurred, adds credence to the approach that was proposed and implemented.

When considering recessive variants that are deleterious in the homozygous state, it is confirmed that these alleles *must* confer some heterozygous survival/fecundity advantage, as recognized by Dubinin and others, otherwise they would never reach noticeable prevalence levels^30^. By using actual prevalence values for a given monogenic variation (such as for CF), and assuming that equilibrium has been reached, one can estimate the selection coefficient, without requiring any knowledge of its specific manifestation and mechanism.

The low probability of even an advantageous allele becoming prevalent, the number of generations it takes to spread through a large population, and the large number (2,000+) of known CFTR variant in humans seem to indicate that the *de novo* mutation rate may be surprisingly high. In addition, from the ExAC database^31^ the CFTR gene seems to harbour a significantly greater number of missense variations with respect to those expected (expected n. missense = 418.8, observed n. missense = 671, missense z-score –6.03).

Most primates as considered by Dunbar and Lehmann admittedly live, mate and die in fairly well-defined troops; humans arguably less so (although in especially prehistoric times there may have been hunter-gatherer bands operating very similarly to current non-human primate groups). However, Dunbar’s number for humans is only used as a reasonable upper value, based on cognitive limitations. It is not claimed that it would necessarily be applicable to other primates or organisms, and even for humans a large variation in group size is admitted and used, exactly because there are so many unknown and environmental factors. Furthermore, the model is not limited to this range, and much of the work presented here explores expected establishment and equilibrium behaviour while varying *N*_n_ over several orders of magnitude, both above and below Dunbar’s number.

## Conclusions

The simulation framework we have developed allows us to create scenarios to study selection and in particular balancing selection, introducing the powerful concept of an effective community size which encompasses the effects of inbreeding and migration, and in which genetic drift and local structure are emergent properties, rather than required inputs which are invariably based on estimates. This could be useful to simulate a realistic scenario of population structure and isolation inside larger populations.

The tool is generally applicable to any monogenic variation in a diploid population, and can be used to estimate the associated selection coefficients, the community size, the establishment probability, or the equilibrium levels. Although the software is admittedly not unique in its simulation capabilities, the simple user interface and highly optimised code facilitates very fast stochastic simulations even when considering huge populations, while running on a standard personal computer.

When specifically considering cystic fibrosis in humans, and assuming equilibrium, we estimate that being a heterozygous CFTR variant carrier confers a selective advantage of at least 3% (depending on our assumption regarding *N*_n_).

The probability of an allele becoming prevalent (i.e. established), as well as the equilibrium level it eventually attains, is strongly linked to the combination of selection coefficient (which includes environment) and the effective community size. While some evidence is presented to support the notion that Dunbar’s number may be a reasonable estimate for the effective community size in some human populations, the tool that we have created allows exploration of arbitrary combinations of selection coefficients, neighbourhood sizes, population sizes, and even mutation rates, although results regarding the latter have not been presented in this paper.

## Methods

A numerical simulation tool was created to facilitate the stochastic exploration of the fate of monogenic variations^32^.

### Assumptions

1. A single genetic locus (with a mutation/variation that changes the procreation probability of individuals) is considered.
2. Compared to the general (wild-type) population, heterozygotes and homozygotes can have different survival/fecundity rates.
3. Individuals select mates for procreation purposes from a limited community *N*_n_, which in general is much smaller than the size of the entire population. *N*_n_ includes the effects of immigration and population structure, obviating the need to estimate these factors.
4. For a population of humans, Dunbar and Lehmann motivate an upper cognitive limit on the number of people with whom an individual can maintain stable social relationships^11,12^. This is used to inform the realistic community size from which an individual can select a mate, in this model. Dunbar’s number for humans is estimated to lie in the range of 100 to 230, with 148 being the nominal value. Values for *N*_n_ of this size and smaller are considered to be reasonable for human populations, although the simulation is not limited to this range.
5. Constant environmental conditions are assumed across the entire population – although provision is made for different geographical conditions to reflect situations such as discussed above - and all simulations were conducted with this assumption of constant conditions in mind.
6. In general, the effective population number *N*_e_ is assumed to be large and comparable to the total population census size *N*), and specifically much larger than *N*_n_, i.e. *N*_e_» *N*_n_. This is also adjustable, however, to allow for exploration of effects in smaller populations and genetic isolates.

### Data Structure

1. A two-dimensional array is created, with every element representing an individual in the population. Computational resources now make it feasible to create and process simulated populations consisting of millions, and even billions, of individuals.
2. Each individual has one of four possible states: dead, wild-type, heterozygous, homozygous.
3. The population array is closed upon itself, with edges wrapping around. This eliminates any edge effects that discontinuities may otherwise introduce.
4. Physical proximity in the elements of the population array is used as a proxy for social closeness – i.e. an individual is more likely to breed with another nearby individual than with a remote one, according to a two-dimensional normal (Gaussian) probability distribution.
5. The effective size of the community around each individual is changed by varying the standard deviation of the normal distribution, with *N*_n_ as in Eq. (1).

### Simulation Procedure

For a given set of parameters, the following steps are executed:

1. Set population size, community size *N*_n_, initial heterozygote prevalence, heterozygous selection coefficient, and *de novo* mutation probability.
2. Initialize the population randomly with a desired initial fraction of heterozygotes.
3. For each individual in the population, change its status to dead with a probability dependent on its current status, to statistically reflect the advantage/disadvantage associated with its status.
4. For each element of the population array:
  - Randomly select two distinct non-dead parents from its community *N*_n_, according to the proximity probability distribution as in Eq. (2).
  - Generate a status according to Mendelian inheritance probabilities from the two parents.
  - Randomly introduce a *de novo* mutation with a specified (typically low and possibly even zero) probability.
5. Update population statistics and display.
6. Return to step 3.

## Supplementary information

S1

S2

S3

S4

## Data Availability

The software, instructions and raw data files used in this study are available on Github with the identifier “10.2581/zenodo. 3257042” ^32^.
