# Author Correction: Establishment and equilibrium levels of deleterious mutations in large populations

**DOI:** 10.1038/s41598-020-62009-8

**Published:** 2020-03-17

**Authors:** Johan W. Viljoen, J. Pieter de Villiers, Augustinus J. van Zyl, Massimo Mezzavilla, Michael S. Pepper

**Affiliations:** 1 Department of Electrical, Electronic and Computer Engineering, EBIT, University of Pretoria, Pretoria, 0028 South Africa; 2 Development, Research and Technology Department, Hensoldt Optronics, Centurion, 0157 South Africa; 3 Radar and Electronic Warfare Research and Applications Group, Council for Scientific and Industrial Research, Pretoria, 0001 South Africa; 4 Department of Mathematics and Applied Mathematics, University of Pretoria, Pretoria, 0028 South Africa; 5 Institute for Maternal and Child Health, IRCCS ‘Burlo Garofolo’, Trieste, Italy; 6 The Wellcome Trust Sanger Institute, Wellcome Genome Campus, Hinxton, Cambridgeshire CB10 1SA UK; 7 Institute for Cellular and Molecular Medicine, Department of Immunology, University of Pretoria, Pretoria, 0084 South Africa; 8 SAMRC Extramural Unit for Stem Cell Research and Therapy, Faculty of Health Sciences, University of Pretoria, Pretoria, 0084 South Africa

Correction to: *Scientific Reports* 10.1038/s41598-019-46803-7, published online 17 July 2019

The original version of this Article contained errors.

Affiliations 1 and 2 were reversed.

Secondly, Affiliation 7 was incorrectly given as ‘Institute for Cellular and Molecular Medicine, Department of Immunology, and SAMRC Extramural Unit for Stem Cell Research and Therapy, Faculty of Health Sciences, University of Pretoria, Pretoria, 0084, South Africa’.

Thirdly, an affiliation was omitted for the author Michael S. Pepper, which is now listed as Affiliation 8.

Fourthly, Affiliation 1 was omitted for the author Johan W. Viljoen.

Finally, Augustinus J. van Zyl was incorrectly affiliated with ‘Institute for Maternal and Child Health, IRCCS ‘Burlo Garofolo’, Trieste, Italy.’

The correct author affiliations are listed below:

Affiliation 1:

Department of Electrical, Electronic and Computer Engineering, EBIT, University of Pretoria, Pretoria 0028, South Africa

Johan W. Viljoen and J. Pieter de Villiers

Affiliation 2:

Development, Research and Technology Department, Hensoldt Optronics, Centurion 0157, South Africa

Johan W. Viljoen

Affiliation 3:

Radar and Electronic Warfare Research and Applications Group, Council for Scientific and Industrial Research, Pretoria 0001, South Africa

J. Pieter de Villiers

Affiliation 4:

Department of Mathematics and Applied Mathematics, University of Pretoria, Pretoria 0028, South Africa

Augustinus J. van Zyl

Affiliation 5:

Institute for Maternal and Child Health, IRCCS ‘Burlo Garofolo’, Trieste, Italy

Massimo Mezzavilla

Affiliation 6:

The Wellcome Trust Sanger Institute, Wellcome Genome Campus, Hinxton, Cambridgeshire CB10 1SA, UK

Massimo Mezzavilla

Affiliation 7:

Institute for Cellular and Molecular Medicine, Department of Immunology, University of Pretoria, Pretoria 0084, South Africa

Michael S. Pepper

Affiliation 8:

SAMRC Extramural Unit for Stem Cell Research and Therapy, Faculty of Health Sciences, University of Pretoria, Pretoria 0084, South Africa

Michael S. Pepper

These errors have now been corrected in the PDF and HTML versions of the paper, and in the accompanying Supplementary Information file.

