# Supplementary material for: Establishment and equilibrium levels of deleterious mutations in large populations
Source: Sci Rep. 2019 Jul 17;9:10384. doi: 10.1038/s41598-019-46803-7 (PMC6637196; doi:10.1038/s41598-019-46803-7)
Supplement: Supplementary file 4 — S4 [file 41598_2019_46803_MOESM4_ESM.zip › Binary Executable/Popsim User manual.docx]

User manual for population simulation code

Supplementary information to manuscript titled:

Establishment and equilibrium levels of deleterious mutations in large populations

**Johan W. Viljoen^1,*^, J. Pieter de Villiers^2^, Augustinus J. Van Zyl^3^, Massimo Mezzavilla^4^, Michael S. Pepper^5^**

^1^Development, Research and Technology Department, Hensoldt Optronics, Centurion 0157, South Africa ([johan.viljoen@hensoldt.net](mailto:johan.viljoen@hensoldt.net))

^2^Department of Electrical, Electronic and Computer Engineering, EBIT, University of Pretoria, Pretoria 0028, South Africa [(pieter.devilliers@up.ac.za](mailto:(pieter.devilliers@up.ac.za))

^2^Radar and Electronic Warfare Research and Applications Group, Council for Scientific and Industrial Research, Pretoria 0001, South Africa

^3^Department of Mathematics and Applied Mathematics, University of Pretoria, Pretoria 0028, South Africa ([gusti.vanzyl@up.ac.za](mailto:gusti.vanzyl@up.ac.za))

^4^Institute for Maternal and Child Health, IRCCS ‘Burlo Garofolo’, Trieste, Italy ([massimo.mezzavilla@burlo.trieste.it](mailto:massimo.mezzavilla@burlo.trieste.it))

^4^The Wellcome Trust Sanger Institute, Wellcome Genome Campus, Hinxton, Cambridgeshire CB10 1SA, UK

^5^Institute for Cellular and Molecular Medicine, Department of Immunology, and SAMRC Extramural Unit for Stem Cell Research and Therapy, Faculty of Health Sciences, University of Pretoria, Pretoria 0084, South Africa ([michael.pepper@up.ac.za](mailto:michael.pepper@up.ac.za))

## Introduction

This software was created to explore the behaviour of monogenic mutations in large populations. It allows independent variation of the selective (dis)advantages of homozygous and heterozygous individuals, as well as the population size, and the community size – that is, the local community from which an individual is likely to select a mate.

## Disclaimer:

Although some effort has been expended to make the software user-friendly and robust, it is by no means bullet-proof; it is quite possible to break it by doing something stupid or clever. This may be due to a mere oversight, incompetence of the programmer, or possibly because of the significant weight given to speed of execution, which may occasionally result in the sacrifice of safety. It has never been the intention to reach commercial levels of polish and refinement.

## Downloading and Deployment

The executable is compiled for use on a PC running Windows 7 and up. It may even work on WinXP, although that has not been tested. Simply place the *.exe file in a suitable folder and run it – no installation required.

## Workflow


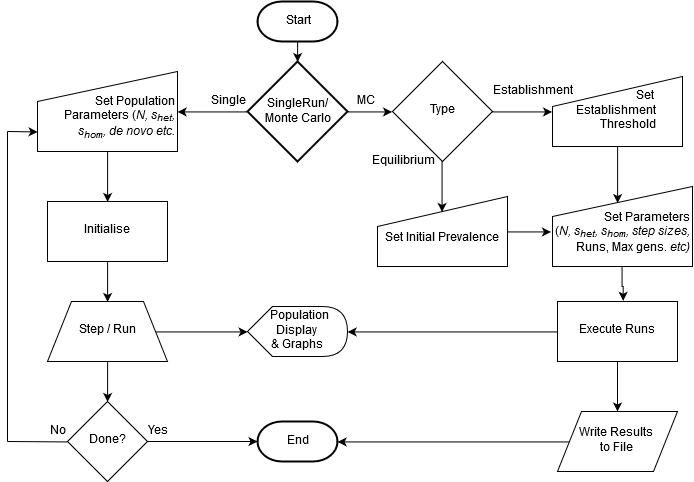


Figure 1 - Basic Workflow diagram

Figure 1 shows the basic workflow when using the simulation software. The various options and functions will be addressed below.

## Main Screen

The program starts by displaying a main screen looking something like this:


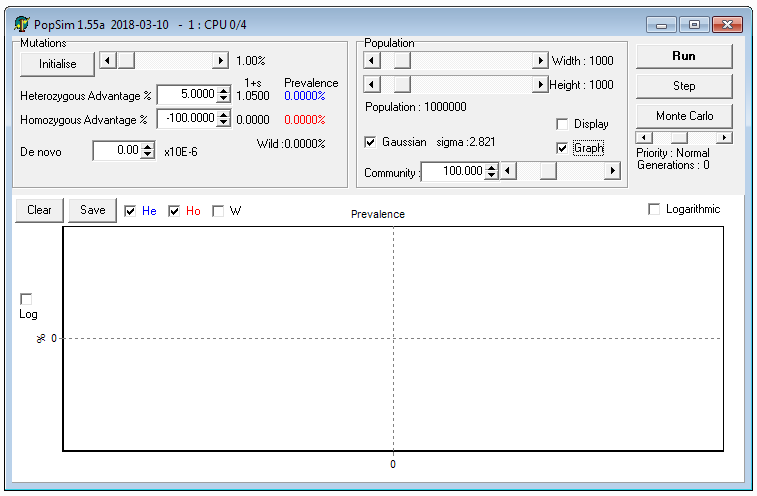


Figure 2 - Main Screen

On the left side, close to the top, there are two text boxes containing respectively the heterozygous and homozygous selective advantages. Note that these numbers are in percentages – to the right of the text boxes is shown the resultant selective advantage; an individual with a selective advantage of *s* will have, on average, *1+s* as many progeny as the wild type. Because it is difficult to have fewer than zero children, these numbers are constrained between -100% and +infinity (or at least an approximation thereof).

On the right, in the panel labelled ‘Population’ the size of the population can be entered – this takes the form of a two-dimensional grid of individuals, wrapping around from top to bottom and left to right to avoid any edge effects. Below these can be found the community size controls: if the ‘Gaussian’ checkbox is selected, the breeding unit (local community) from which an individual is likely to select a mate takes the shape of a two-dimensional Gaussian distribution, with an effective size that of a circle with radius 2σ. Nearby individuals are more likely to be selected than remote ones. If ‘Gaussian’ is unchecked, the community is a circle with size (in individuals) as shown, with all included individuals equally likely to be selected (i.e. a flat distribution).

When the ‘Display’ checkbox is checked, the population will be displayed:


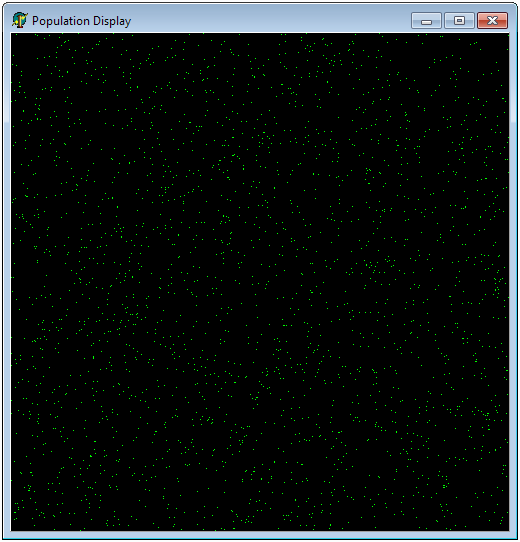


Figure 3 - Population Display

In this specific case the population was initialised to 1% heterozygous prevalence by clicking on the ‘Initialise’ button at the top left, with the slider to the right of it set to 1%, of course. Heterozygous individuals are shown as green dots, homozygous ones (of which there are none in this example) as white dots, while the wild type is shown in black.

Clicking on ‘Step’ will now advance the simulation by one generation, using the selective advantages and community size settings as selected. If the ‘Graph’ checkbox, just below ‘Display’ is checked, the graph at the bottom of the main screen will also be updated with the relative prevalence numbers of the Homozygous, Heterozygous and Wild Type individuals. These graphs can be individually switched on or off by checking the applicable checkboxes at the top left of the graph.

Clicking on ‘Step’ soon becomes wearisome, so click on ‘Run’ at the top right of the main screen. This will keep stepping through generations at maximum speed until you hit the same button again (which should be labelled ‘Stop’ while the simulation is running).

Do note that having the population display active will in general slow the simulation down significantly. It takes time to update the pixels. Not much, but there are often many of them. Also, especially when the population size exceeds that of the actual screen, things may become slightly unstable: to speed things up the screen is updated by directly addressing the video memory, which sometimes leads to unwanted effects when the display extends off-screen.

Most of the parameters can be adjusted while the simulation is running – this can be used to explore interactively.

## Monte Carlo analysis

Because it quickly gets tedious to keep adjusting parameters, a Monte Carlo function was created to automate this process too. Click on the ‘Monte Carlo’ button:


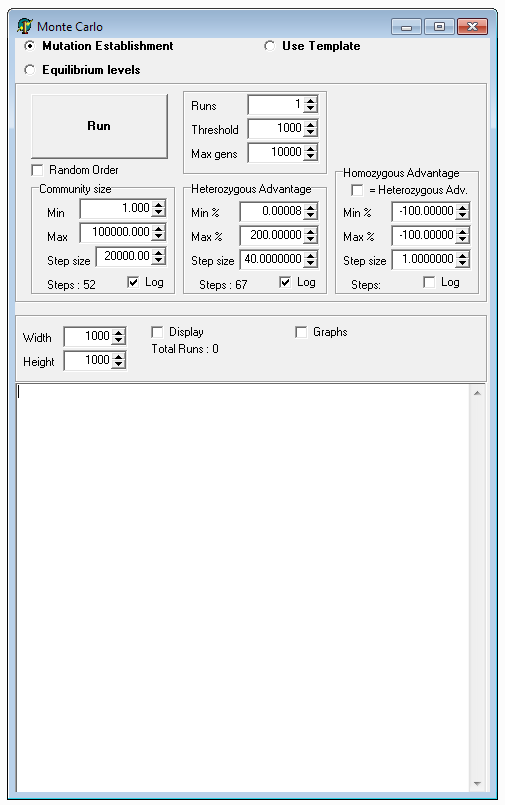


Figure 4 - Monte Carlo screen

The very first choice, at the top, is between ‘Mutation Establishment’ and ‘Equilibrium Levels’.

### Mutation Establishment

When ‘Mutation Establishment’ is chosen, the screen will look something like shown in Figure 4.

A run involves creating a population sized as shown at the bottom left, initialised to wild type for all individuals, and then inserting a single instance of a mutation (i.e. one heterozygous carrier) into the population. After this, the simulation is run using the selected parameters until one of the stopping criteria is reached.

Multiple simulations are executed sequentially. To prevent any given run to continue indefinitely, there has to be stopping criteria. These are: Extinction, Prevalence and Time.

- Extinction: If the mutated gene becomes extinct, the simulation can be stopped, for nothing else will happen after that point.
- Prevalence: A mutation that does not become extinct tends to grow in prevalence. A threshold is set which, if exceeded, is considered to constitute evidence that the mutation has gained sufficient traction to make extinction unlikely, i.e. it has become established. This is set in the ‘Threshold’ text box at the top of the Monte Carlo screen.
- Time: It may happen that a mutation manages to linger in the population at very low prevalence levels (below the threshold set above), yet not become extinct – to prevent such situations from locking up the simulation, an upper limit on the number of generations is set in the text box labelled ‘Max gens’.

### Community Size

The community size can be automatically stepped from the value set in the ‘Min’ box to the value in the ‘Max’ box, with increments as set in the ‘Step Size’ control below it. If the ‘Log’ checkbox is checked, the community size will be changed not linearly, but logarithmically, with the final step the size of the value in the ‘Step Size’ control. The way this is done is to start at the maximum value, then decrement by ‘Step Size’, and for each subsequent run keep decrementing by that same *ratio,* until reaching the minimum value.

### Heterozygous Advantage

This parameter can also be changed automatically, in the same way as the community size. Understandably the logarithmic function does not work so well when negative values are desired.

### Homozygous Advantage

This parameter can also be automated. When the ‘= Heterozygous Adv.’ option is selected, it will be linked to the heterozygous advantage, with the same value being used.

Multiple runs with identical parameters can be executed by specifying the number of repetitions in the top middle, in the box labelled ‘Runs’.

When a run is completed, a summary of the run is displayed in the panel at the bottom of the Monte Carlo screen. This same information is also written to a text file for later analysis. The file name starts with ‘MC’, followed by the start time and date, and will be found in the same folder as the program itself.

During Monte Carlo runs it is also possible to activate the prevalence graphs, or, even worse, the population display. This is rarely a good idea if completion time is important, as it often is.

## Equilibrium Levels


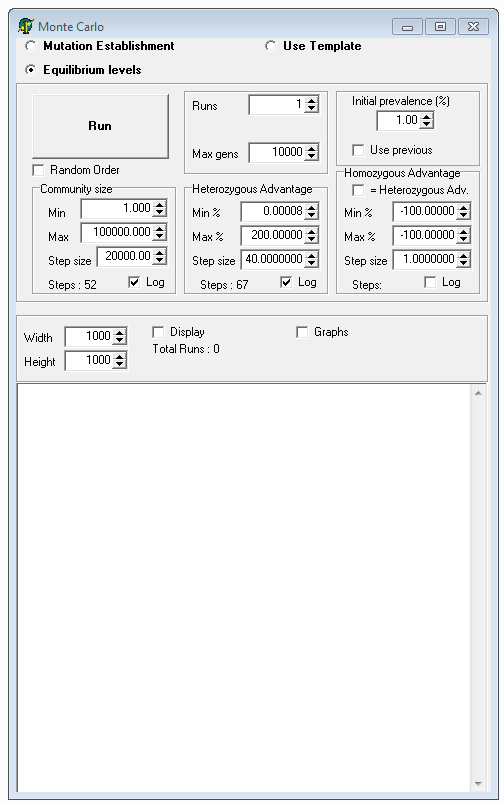


Figure 5 - Equilibrium levels

When the ‘Equilibrium levels’ option is selected, the Monte Carlo screen changes to something like Figure 5. Most controls function similarly to the Mutation Establishment case, except that, instead of placing a single instance of a mutation in the middle of the population at the start of each cycle, the population is randomly initialized to the prevalence specified at the top right (clearly labelled ‘Initial prevalence (%)’).

The stopping criteria are Extinction, Prevalence, Time and Stability:

- Extinction: If the mutated gene becomes extinct, the simulation can be stopped, for nothing else will happen after that point.
- Prevalence: A mutation that does not become extinct tends to grow in prevalence. The simulation is stopped when 100% is reached as this is actually identical to the Extinction case above, as seem from the wild type’s perspective.
- Time: It may happen that a mutation manages to linger in the population at intermediate prevalence levels, yet not become extinct – to prevent such situations from locking up the simulation, an upper limit on the number of generations is set in the text box labelled ‘Max gens’. This is unlikely to happen though, because of the next item:
- Stability: If the simulation detects that the long-term prevalence levels have stabilised (somewhere between 0% and 100%, both excluded), the simulation is terminated. Due to genetic drift there is always some variability in the levels. The code for this is fairly conservative, to reduce false triggers prematurely terminating a run.

## Use Template

At the start of each Monte Carlo run a template file is created, containing a summary of the run. This is to simplify repetition of an entire run. This file can be used when the ‘Use Template’ option is selected – in that case a button named ‘Template File’ appears, which is used to select the relevant template file. Do note that the option exists to also use the normal result files (starting with ‘MC’) as a template – it may just take slightly longer to analyse if it contains many repetitions of each run.

## Processor control

Monte Carlo runs can easily take very long to complete, especially when the population size is large and/or many runs are desired. Some effort was expended to ensure maximal utilisation of processor resources.

During startup the program determines the effective number of processor cores in the host computer, and sets its affinity to one of these. This information is displayed in the title bar at the top of the main screen. In the example shown in Figure 2, the program finds itself on a four-core machine, and assigned itself to core 0 of those 4 (number 0, silly as it sounds, is actually the first core, because that is how some people, including Intel, counts). If a second instance of the program is started, it will run on the next available processor core (1, in this case) etc. This is to ensure that the program does not compete with itself for processor resources, overriding the control exerted by Windows, which will happily let many processes run on one core, while others remain essentially idle. If more instances of the program are run than there are processor cores, they will of course start at zero again, and be forced to share. This is probably not a good idea.

Additionally, the priority of each process can also be controlled using the slider at the right of the main screen, just below the ‘Monte Carlo’ button. This is to enable peaceful co-existence with other programs and users, especially when all cores are being utilised by instances of this simulator. Setting the priority to ‘Below Normal’ or even ‘Idle’ means that the PC should still remain responsive, and useful for other work, even while all cores are kept 100% busy.

The above can of course also be done using the Windows Task Manager, but this way usually saves time and limits mistakes.
